# A Realist Inquiry to Identify the Contribution of Lean Six Sigma to Person-Centred Care and Cultures

**DOI:** 10.3390/ijerph181910427

**Published:** 2021-10-03

**Authors:** Seán Paul Teeling, Jan Dewing, Deborah Baldie

**Affiliations:** 1 UCD Centre for Interdisciplinary Research, Education and Innovation in Health Systems, School of Nursing, Midwifery & Health Systems, University College Dublin, Dublin D04 V1W8, Ireland; 2 Mater Lean Academy, Mater Misericordiae University Hospital, Eccles Street, Dublin D07 R2WY, Ireland; 3 Centre for Person-Centred Practice Research Division of Nursing, School of Health Sciences, Queen Margaret University Drive, Queen Margaret University, Musselburgh, East Lothian, Scotland EH21 6UU, UK; JDewing@qmu.ac.uk (J.D.); DBaldie@qmu.ac.uk (D.B.); 4 Nursing and Midwifery Directorate, NHS Grampian, Scotland AB25 2ZN, UK

**Keywords:** Lean, Six Sigma, Lean Six Sigma, process improvement, person-centredness, person-centred care, person-centred cultures, realist evaluation

## Abstract

A lack of fidelity to Lean Six Sigma’s (LSS) philosophical roots can create division between person-centred approaches to transforming care experiences and services, and system wide quality improvement methods focused solely on efficiency and clinical outcomes. There is little research into, and a poor understanding of, the mechanisms and processes through which LSS education influences healthcare staffs’ person-centred practice. This realist inquiry asks ‘whether, to what extent and in what ways, LSS in healthcare contributes to person-centred care and cultures’. Realist review identified three potential Context, Mechanism, Outcome configurations (CMOcs) explaining how LSS influenced practice, relating to staff, patients, and organisational influences. Realist evaluation was used to explore the CMOc relating to staff, showing how they interacted with a LSS education Programme (the intervention) with CMOc adjudication by the research team and study participants to determine whether, to what extent, and in what ways it influenced person-centred cultures. Three more focused CMOcs emerged from the adjudication of the CMOc relating to staff, and these were aligned to previously identified synergies and divergences between participants’ LSS practice and person-centred cultures. This enabled us to understand the contribution of LSS to person-centred care and cultures that contribute to the evidence base on the study of quality improvement beyond intervention effectiveness alone.

## 1. Introduction

### 1.1. Background

In this paper, we undertake a realist inquiry to identify and to understand the contribution of Lean Six Sigma (LSS) to person-centred care and cultures, an under researched area. We detail the first study to use realist evaluation to explore the degree and nature of the coherence between LSS and person-centred methodologies in healthcare practice. In this first section, we introduce the process improvement methodology of LSS in relation to its component parts, Lean and Six Sigma. We then outline the concepts of person-centred care and person-centred cultures. Finally, we set out the problem that the study seeks to address.

#### 1.1.1. Lean Six Sigma

LSS is a combination of two process improvement methodologies, Lean, developed by Toyota, and Six Sigma, developed by Motorola [1]. Lean has been described as a quality improvement approach that consists of the elimination of waste (steps that do not add value in the eyes of the customer) to improve the flow of people, information, or goods [2,3]. Six Sigma is a data-driven process improvement methodology designed to improve process capability and enhance process throughput through the introduction of improvement projects [4,5,6]. A hybrid of Lean and Six Sigma as LSS appears in the healthcare literature from 2010 onwards [1] following Lean and Six Sigma integration for project delivery from early 2002 and increased use by 2008. Both methodologies have a strong focus on the customer, the employee, management support, and teamwork [7,8,9]. LSS has evidenced improvement in healthcare settings at patient, staff, and organisation levels [10,11,12,13,14]. Lean, Six Sigma, and LSS are now considered to be some of the most popular process improvement methodologies used in healthcare internationally [15,16,17,18].

#### 1.1.2. Person Centredness

Simultaneous to the development and use of LSS in healthcare, political and policy stakeholders have widely advocated that person-centred care should be at the heart of the health system [19,20,21,22,23]. Person centredness refers to embedded practices within a specific type of culture that enables and facilitates the delivery of person-centred care [24]. Person-centred cultures are deemed necessary for the delivery of person-centred care [25]. Person-centred care has an explicit focus on ensuring the client or patient is at the centre of care delivery [26] and is concerned with every person involved in the patient’s care, not just the patient [25,27,28]. McCormack notes that healthcare can often rely exclusively on measurement (metrics), hard evidence, and tangible outcomes, which are not necessarily facilitators of person centredness [29]. McCormack and colleagues [30] state that healthcare delivery needs to move towards a culture that contextualises and integrates the ‘hard’ evaluation of outcomes and programmes within an overarching person-oriented evaluation framework. This study builds on our previous work that identified synergies and divergences between LSS and person-centred methodologies that support the enhancement of person-centred cultures, which can reliably provide person-centred care [18].

#### 1.1.3. The Problem

Increasingly, healthcare organisations internationally are using both LSS and person-centred methodologies in the planning and delivery of care [18]. LSS has a founding principle on valuing the person or customer and ensuring that products or services are designed around their specific needs. This principle is one that is gaining increasing traction within modern health care design and redesign [18]. Whilst there is a wide body of research on LSS demonstrating its impact on effectiveness and efficiency, there is little known about the influence of LSS on person-centred care and person-centred cultures, and the relationship between them. Considering person-centred culture is seen as a prerequisite of person-centred care practice [26], it is important to understand what relationship LSS has to person-centred cultures and what potential health care benefits it might offer. Dixon Woods [31] suggests that if we are to improve health services, then the study of quality improvement methodologies in healthcare is essential. Dixon Woods [31] further argues that such work is critical in developing an evidence base that looks at more than interventions alone but also at the way they are introduced and evaluated in practice, i.e., the effectiveness of quality improvement approaches. This study therefore aimed to contribute to the evidence base for quality improvement approaches by addressing a gap in the evidence relating to LSS. We aimed to evaluate the influence LSS, as taught on a university education and training programme, had on person-centred cultures in participants’ practice areas, and to explore what health care benefits that could lead to.

#### 1.1.4. Study Aim

The aim of this study was to address the question: whether, to what extent, and in what ways do Lean and Six Sigma in healthcare contribute to person-centred care and cultures? The objective was to understand how healthcare staff who are LSS practitioners and have undertaken a university LSS education and training programme understand and experience, in their specific contexts of practice, the contribution of the application of LSS learning and practice to person-centred care and cultures. This will contribute to knowledge of how LSS and person centredness can be used together in future process improvements.

## 2. Materials and Methods

### 2.1. Study Design

#### 2.1.1. Study Setting

The study site was a university teaching hospital in Dublin, Ireland, a major centre for medical, nursing, and allied health professional training and a teaching partner to University College Dublin (UCD) since its foundation. Between 2014 and 2020, a LSS staff education and training programme, a joint undertaking between UCD and the study site [32], has delivered over 200 process and quality improvement projects in over 50 healthcare institutions and Community Healthcare Organisations (CHOs) in Ireland. LSS projects were undertaken to improve service delivery from the perspectives of both patients and staff.

#### 2.1.2. Study Design

The study design used realist inquiry, which seeks to develop a common understanding of underlying factors and causative mechanisms and, according to Pawson [33], seeks to understand the components of the social world and stratifications of social reality. Traditional orthodox research strategies, such as systematic reviews or evaluation studies, look for the answer to the question ‘what works?’ In realist inquiry there is a focus not only on ‘what works’ but on ‘what works for whom, why it works, and in what circumstances’ [33,34]. From a realist inquiry viewpoint, a CMO configuration (CMOc) can be seen as a hypothesis that a programme outcome (O) emerges because of the action of underlying mechanisms (M), which are activated when participants engage with the intervention and such mechanisms are influenced by the particular contexts the intervention is placed within (C). Pawson [33] sees social programmes as providing resources that activate people’s reasoning and actions: the mechanism (M). However, Pawson [33] states that the activation of the mechanism is dependent on variables, such as individual characteristics, circumstances, and situations: the context (C), which leads to variation in how they interact with the intervention and thus what outcomes (O) are achieved. This approach to evaluating social programmes enables the theories within a programme to be made explicit, by developing clear hypotheses about how, and for whom programmes might work, and in what context. For Pawson and Tilley [35], CMOcs are the foundation upon which all realist understanding is built. They also propose that CMOcs are iterative, and it is this iterative process that brings together and captures variations in both mechanisms and contexts; as the mechanism is “fired”, the context is changed, which further influences subsequent mechanisms and the exploration of an intervention’s impact through this lens enables researchers to understand and attempt to predict and explain outcome pattern variation.

**Figure 1:**
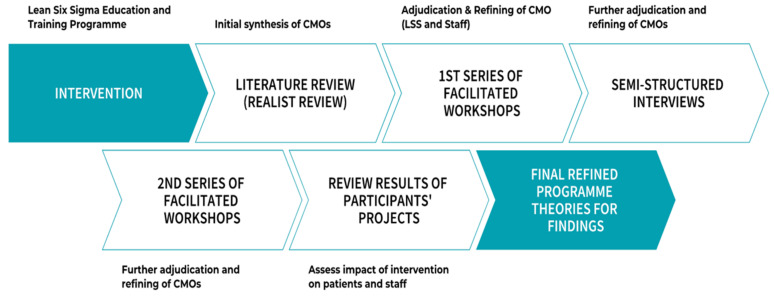
Methodological process.

Within realist inquiry, two approaches have come to the fore: realist review and realist evaluation. Realist review, sometimes known as realist synthesis, is a companion research approach to realist evaluation, involving the analysis of existing data including stakeholders’ views and opinions, whilst realist evaluation is a form of inquiry utilised for primary research and involves the collection of data from the source [35,36,37,38,39,40,41,42]. Realist evaluation focuses on interventions and there is a growing body of work using realist inquiry to analyse interventions within healthcare organisations [43,44] including those using LSS methodology [12,45,46]. In this study, the intervention was identified as the university-delivered LSS staff education and training programme. Realist evaluation is a theory-based evaluation designed to test and refine a theory that has informed the development of programmes or interventions. Commensurate with a critical realist approach [47], this study used both realist review to develop programme theory and realist evaluation to collect primary data to test and further refine it. Figure 1 indicates the key research steps during this study.

Generally, realist evaluations begin with an initial programme theory (hypothesis) and end with a more developed theory [48]. The first step in conducting realist evaluation is, therefore, to develop the programme theory to explain how the proposed intervention (the LSS education and training programme) is expected to work in the eyes of the programme designers and implementers [35].

#### 2.1.3. Reference Group

Congruent with realist evaluation, the development of the initial programme theory was guided by a core expert group [49]. This group included an LSS healthcare practitioner, with an MSc level specialist qualification and 10 years’ experience of quality and process improvement using LSS in healthcare settings. It also included a university lecturer in LSS use in healthcare who designed and lectured on LSS programmes. Two experts in person-centred practice research on person-centred cultures and care completed the core group. Additional expertise was provided by a post-doctoral researcher in person-centred practice research and person-centred cultures and care, and a senior nurse in research and practice development who was experienced in person-centred principles and processes and realist evaluation.

#### 2.1.4. Initial Programme Theory Development

Based on the expertise of the reference group and the literature on LSS and person-centred methodologies, we discussed a set of potential explanations as to how the intervention of LSS might contribute to person-centred care and cultures. This drawing on the expertise of the reference group led to the development of an initial, high level, programme theory: LSS can have a positive influence on person-centred care and person-centred cultures if delivered through the intervention of the LSS staff education and training programme. We initially tested this through a realist review.

#### 2.1.5. Realist Review

The databases CINAHL, EBSCOhost, Proquest, and Medline were initially used to identify studies that were relevant to the review questions and involved LSS, person-centred care, and person-centred cultures or a combined use of both LSS and person-centred care methodologies in healthcare. The studies encompassed both empirical and conceptual work. Opinion or editorial pieces were excluded. Three strands were identified for the literature search:

1. Key search terms used in combination for the first strand were: ‘Lean’, ‘Six Sigma’, ‘Lean Six Sigma’, ‘process improvement’, ‘quality improvement’, and ‘healthcare’.
2. The second strand search utilised the key words ‘person-centred’, ‘person-centredness’, ‘person-centred cultures’, ‘patient-centred’, and ‘patient-centredness’.
3. The third strand of the search utilised a combination of the keywords from strands one and two to fully address the research questions.

Across all three strands, reference lists of retrieved articles were examined for the key search terms in their titles and affiliated searching of the reference lists of retrieved items was also conducted to identify further research articles not identified through the keyword searches. The following inclusion and exclusion criteria were applied in order to narrow the search results:

1. Work published relating to LSS in healthcare in the English language between 2000 and 2017, the rationale for this being that LSS was first introduced into healthcare settings early in the decade.
2. Work relating to LSS that discusses the concept of patient-centred care but also references person-centred care or person-centred cultures.
3. Work relating to person-centred care and person-centred cultures from 1995 to 2017, the rationale being that this was a period of ‘strong academic momentum and practice emergence’ [50].
4. Work meeting criteria 1–3 that was peer-reviewed and available as full-text journal articles with a complete bibliography.

**Figure 2:**
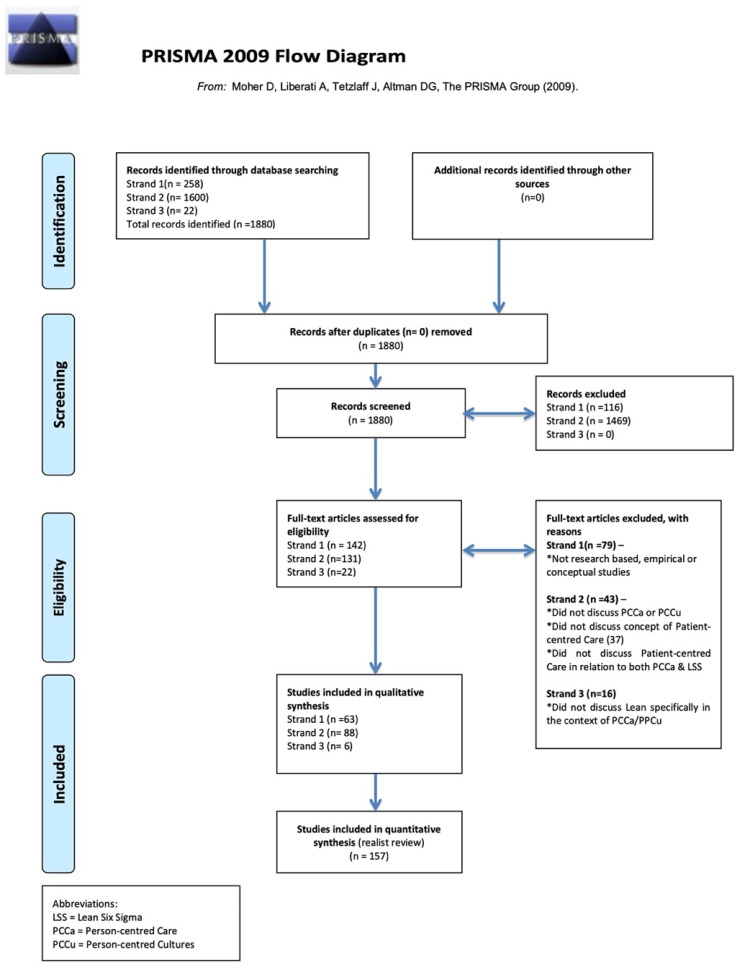
Modified Prisma diagram.

Our inclusion criteria facilitated a comprehensive and high-quality yield of papers (Figure 2) for realist review, a theory-driven and interpretive type of literature review [35,36,37,38,39,40,41,42], up to and including studies published by September 2017. This review led to the development of three potential Context, Mechanism, Outcome configurations (CMOcs) that explained how LSS influenced practice, relating to patients, staff, and organisational influences. In reviewing the three CMOcs, it became evident that within the scope of the continuation of this study, it would not be possible to address all three through empirical realist evaluation. Revisiting the review of the literature, the reference group decided to focus on the CMOc relating to staff. The rationale for this focus were as follows:

1. The outcomes for the CMOc relating to patients were dependent on the CMOc relating to staff, in that staff proficiency and knowledge of LSS are mechanisms for patient outcomes.
2. The CMOc relating to organisational influences addressed organisational support; however, the CMOc relating to staff addressed where and how staff worked with LSS in their everyday practice. To truly capture the staff voice, the logical choice for further research was the CMOc that related to them.
3. Much of the literature talked about organisational gains in relation to Key Performance Indicators (KPIs) but little referred to how staff felt about their interactions with LSS. As this study was based on the concept of person-centred cultures, there was a need to focus this research on staff as a key component of the impact of LSS.
4. There was a large population of staff who were trained in, led and worked on LSS initiatives within the study site, and who were willing to participate in the study.

The CMOc relating to staff was therefore the focus of further adjudication through realist evaluation.

#### 2.1.6. Realist Evaluation

**Figure 3:**
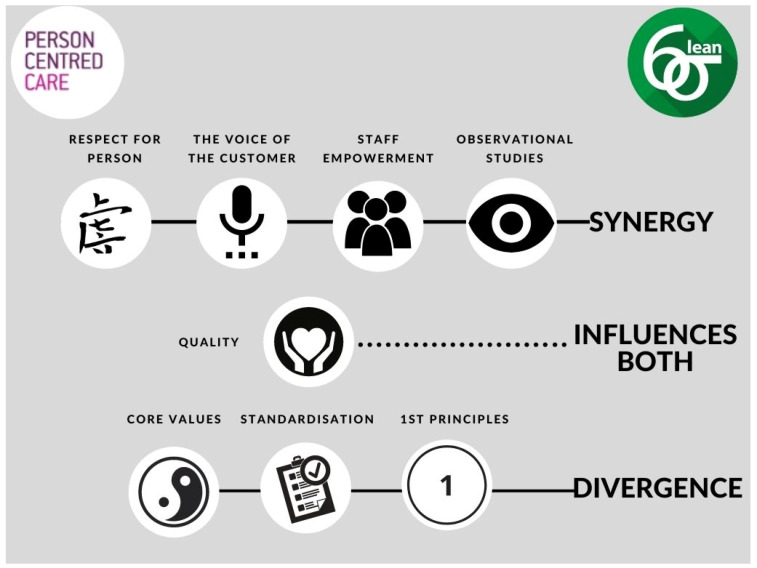
Synergy and divergence between LSS and person-centred care (Teeling, Dewing & Baldie, 2020).

We sought to understand the influence of LSS on staff through exploring staffs’ lived experiences of engaging with the programme and using the data generated to, collectively with research participants, adjudicate the theory and evidence-informed CMOc. In realist evaluation, the use of a broad range of data is seen as increasing the robustness of the process of theory building and testing [47]. Data is required that can identify and elucidate Contexts, Mechanisms, and Outcomes, and inform the relationships between them. Data collection was completed in iterative stages using a combination of data collection methods [35]. These are represented in Figure 1. To facilitate this, following the realist review, further data collection was carried out in the following sequence:

1. A series of facilitated workshops with study participants (n = 20) to adjudicate the CMOc, relating to staff. The person-centred principles of Collaborative, Inclusive, and Participative (CIP) ways of working [51,52] underpinned the approach to these workshops to gather participants’ views and experiences as LSS practitioners. A range of creative approaches were used to achieve these collaborative and inclusive ways of working including the use of pictures and creative constructs [53] and other means, such as the use of painting and collage [54,55]. These approaches facilitated participant feedback and adjudication of the programme theory, adjudication being the interrogation of underlying causal processes [40], and further facilitated thematic analysis. The ultimate purpose of data analysis through adjudication is related to theory development and refinement of the programme theory [56].
2. Individual interviews with workshop participants (n = 20) to further explore the themes that were developed in their first workshop and to refine the initial programme theory through individual adjudication of the CMOc. These were semi-structured realist interviews [57].
3. A second series of facilitated workshops with study participants to arrive at a final adjudication of the CMOc as refined in workshop one and further refined in the individual interviews. Participants worked with the researchers to consider the data from the realist review, from LSS poster presentations and from their own workshop and individual interviews and to use that information to adjudicate the existing CMOc. Finally, participants located the adjudicated CMOc within the synergies and divergences identified between LSS, person-centred care, and person-centred cultures located in a previous review of the literature by the authors of this study [18]. These synergy and divergences are represented in Figure 3.
4. The results of study participants’ LSS projects, already in the public domain, provided supporting evidence for the outcomes of their LSS work in their area of practice and provided evidence of improved patient and staff experiences, and patient outcomes. Results reviewed were represented in scientific poster presentations and peer-reviewed publications.

Data collection took place over a seven-month period between January and July 2019.

#### 2.1.7. Study Participants

The sample size pragmatic (n = 20), derived from study site staff from a range of disciplines and functions, all graduates of a LSS education and training programme, having graduated between 2014 and 2017, and selected from those currently working in the hospital (n = 97). This sample constituted 20% of the population of LSS graduates in the study site and was a feasible number of participants to work with. Graduates of the intervention programme were chosen in line with realist evaluation principles, with Pawson and Tilley [35] highlighting that they will probably have experienced both the successes and failures of the programme intervention and will be best placed to advise on outcomes. Participants were selected by purposive sampling according to the following criteria:

Inclusion criteria:

- Graduate of the LSS education and training programme (2014–2017).
- Current member of staff within the study site.

Exclusion criteria:

- Graduates of the LSS education and training programme (2014–2017) who no longer worked in the hospital (as their LSS practice did not relate to the study site).
- Non-study site staff who undertook the programme.
- Graduates currently involved in another research project (so as not to detract from their work on that project or place additional demands on their time).

The use of purposive sampling was designed to enable data generation on the study’s programme theory, draw clear inferences and credible explanations from the data that was generated, and to be as efficient as practical [58]. Participants identified different contexts (C), which, with key mechanisms (M), were considered to trigger/prevent a range of outcomes (O) where the intervention of the LSS education and training programme was introduced. Consistent with realist evaluation methodology, participants confirmed, refuted, or refined the programme theory [35] and identified what, in their experience, facilitated or hindered the effectiveness of the intervention to deliver anticipated outcomes [59].

## 3. Results

### 3.1. Results of the Realist Review

**Figure 4:**
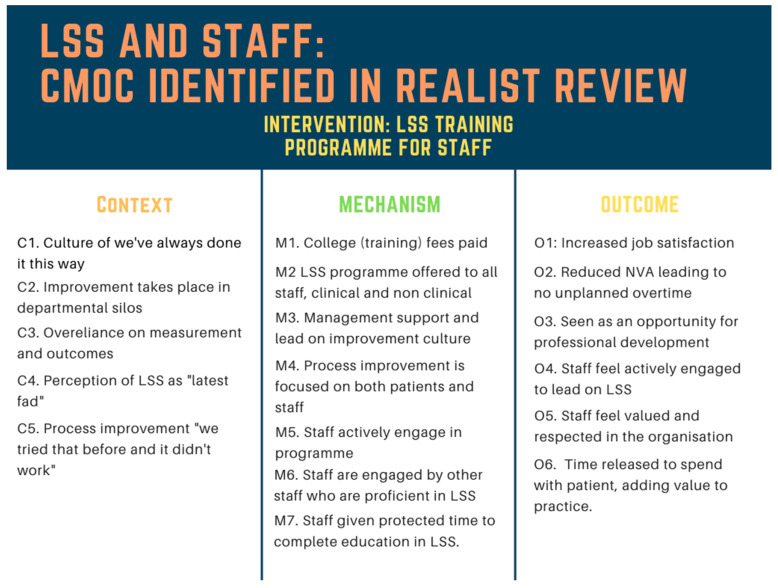
CMOc extracted from the realist review relating to LSS and staff.

The CMOc relating to staff extracted from the realist review identified five potential contexts or contextual factors, seven mechanisms, and six outcomes (Figure 4). The CMOc identified that staff who were LSS practitioners often operated in contexts (C) where they met expressions of ‘we’ve always done it this way’ (C) [60,61] with improvement happening in silos and not across departments or organisations (C) [3,9,10]. They also encountered perceptions of LSS as ‘the latest fad’ (C) [62,63,64] and feelings of ‘we tried that before and it didn’t work’ amongst colleagues(C) [65]. Another context they encountered was that healthcare staff often expressed that there was an overreliance on measurement and outcomes [18,26,29]. Mechanisms (M) that facilitated engagement with LSS interventions included providing staff supports for education and training in LSS [66,67,68,69] including assistance with fees (M) and protected time to complete education (M). The interdisciplinary nature of LSS enabled staff across all disciplines [70] to access LSS education and training programmes (M), actively self-selecting for them (M), and finding support in their practice areas from other staff already proficient in LSS (M) [71]. The presence of management who supported and led on an improvement culture (M) [66] further encouraged staff participation in LSS interventions [1,72], particularly where improvement was focused on both patients and staff (M). Outcomes (O) identified when staff engaged with LSS interventions included increased job satisfaction [73,74,75], reduction in staff overtime [13,76,77,78], and time released to care [79,80,81]. Staff felt that as LSS practitioners they were valued and respected in their organisations (O) [18,82,83], actively engaged to participate, and lead on improvement (O) [9,84,85,86] and that they further saw LSS qualifications as an opportunity for professional development (O) [13,76,77,78].

Having developed the CMOc relating to staff (Figure 4) in the realist review, our aim was to further explore this to identify and understand LSS’s contribution to person-centred care and person-centred cultures through empirical testing of the programme theory using realist evaluation.

### 3.2. Results of the Realist Evaluation

Study participants’ review and refinement of the CMOc relating to LSS and staff extracted from the realist review (Figure 4) led to the development of three new and more focused CMOc, each of which related to LSS and staff. These were:

1. CMOc 1. LSS and aspects of organisational culture;
2. CMOc 2. The organisation’s receptivity to LSS;
3. CMOc 3. Participants’ self-perception as LSS practitioners.

Following the development of the three new CMOC, participants further aligned each of the individual CMOc to the previously identified synergy and divergence between LSS and person-centred care [18] (Figure 3). Each of these CMOc and their alignment to the synergy and divergence model (Figure 3) is now outlined.

#### 3.2.1. CMOc 1. LSS and Aspects of Organisational Culture

At the study site, LSS practitioners had experienced LSS as an integrative and distributive approach (C) that was well communicated to staff (C) who were open to new ways of working (C). The study site also had a high number of competent LSS practitioners (C). Management within the hospital were visibly active in their support of LSS students and practitioners (M) who used a project charter template to focus their improvement work (M). The promotion of LSS at the departmental level (M) ensured it was understood within specific practice areas and staff were aware of, and used, the support available from the on-site service improvement team (M). In the organisation, staff engagement with LSS led to a focus on staff experience in addition to that of patients (O) and an increase in the quality of patient care and outcomes (O). LSS had also led to a recognised change in the organisational culture (O) and to an ability for staff to transcend their traditional practice area silos (O). In situations where LSS practitioners had in the past encountered a reaction of ‘we’ve always done it this way’ (C) or ‘we’ve tried that before and it didn’t work’ (C) or where improvement took place in silos (C), LSS practitioners indicated how that had adversely influenced their engagement with the LSS education and training programme, and that the anticipated outcomes (O) were not achieved.

**Figure 5:**
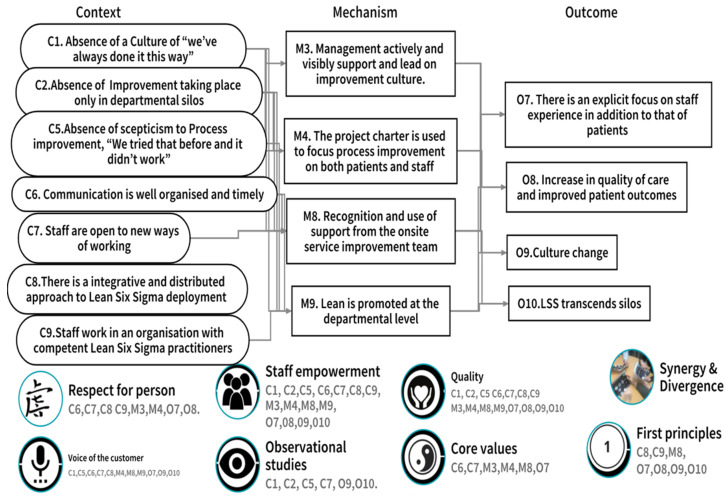
CMOc1 LSS and aspects of organisational culture.

Figure 5 illustrates the contextual factors (Context), Mechanisms, and Outcomes that were confirmed, refuted, or refined through participant adjudication [35]. The previously identified synergy and divergence between LSS and person-centred care [18] are also mapped to the bottom of Figure 5. Finally, each of the noted synergy, divergence, or influence, relating to CMOc1 ‘LSS and aspects of organizational culture’, are numbered and therefore associated to the relevant Context, Mechanism, and Outcome. This allows us to visualise the alignment of CMOc1 to the synergy/divergence model (Figure 3) and reflects where study participants, through CMOc adjudication, aligned individual contexts, mechanisms, and outcomes to identified synergy, divergence, or influencer.

#### 3.2.2. CMOc 2. The Organisation’s Receptivity to LSS

LSS practitioners’ experiences at the study site indicated that improvement and change were achievable (C). They also found that the resourcing of practice areas (C) was important for process improvement to occur. The payment of college education and training fees (M) and protected time for staff (M) influenced their engagement with the LSS education and training programme. Further protected time (M) to carry out improvement work was identified as important for staff who were seen to actively engage in and self-select (M) for the LSS education and training programme. Finally, the availability of the education and training programme to all staff (M) was seen as being important for staff engagement with LSS. Where the identified contextual factors (C) triggered the mechanisms (M), outcomes (O) included a perception of the education and training programme as an opportunity for professional development (O) that led to time released to spend with their patients (O). Additionally, these contexts and mechanisms facilitated staff working in collaborative inclusive and participatory teams (O) that saw LSS projects as platforms for continuous improvement. In contexts where there was an overreliance on measurement and outcomes (C), LSS practitioners indicated that it had a negative influence on their understanding and interpretation of improvement, and consequently their engagement with the intervention of the LSS education and training programme and the anticipated outcomes (O) were not achieved.

**Figure 6:**
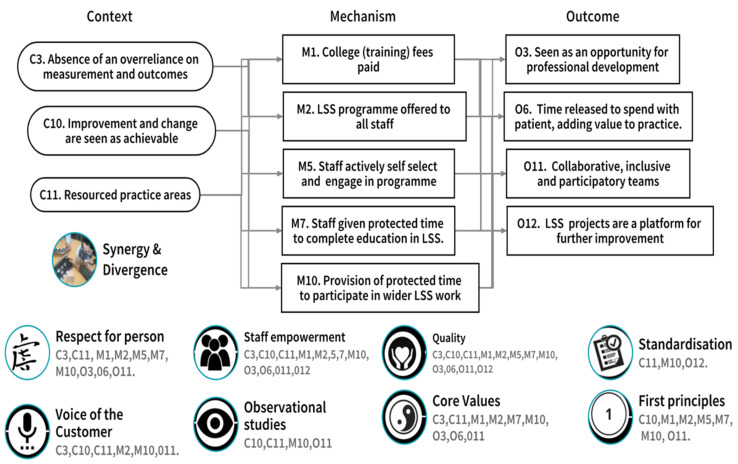
CMOc2 The organisation’s receptivity to LSS.

Figure 6 illustrates the contextual factors (Context), Mechanisms, and Outcomes that were confirmed, refuted, or refined through participant adjudication [35]. The previously identified synergy and divergence between LSS and person-centred care [18] are also mapped to the bottom of Figure 6. Finally, each of the noted synergy, divergence, or influence, relating to CMOc2 ‘The organisation’s receptivity to LSS’, are numbered and therefore associated to the relevant Context, Mechanism, and Outcome. This allows us to visualise the alignment of CMOc2 to the synergy/divergence model (Figure 3) and reflects where study participants, through CMOc adjudication, aligned individual contexts, mechanisms, and outcomes to the identified synergy, divergence, or influencer.

#### 3.2.3. CMOc 3. Participants’ Self-Perception as LSS Practitioners

The study site did not use external consultants to lead on improvement work but rather internal staff who were LSS practitioners (C). The organisation viewed these staff as leaders in their ongoing process improvement work (C). LSS practitioners engaged with staff who were receptive to them (M) and who were encouraged to engage in LSS improvement work by the dissemination of the results of previous work (M) relating to patient and staff experiences of care, and patient outcomes. Peer support from colleagues and other LSS practitioners (M) was also seen as an important factor in supporting practitioners in their work. Where the identified contextual factors (C) triggered the mechanisms (M), outcomes included staff feelings of increased job satisfaction (O), staff feeling valued and respected, and engaged to lead on improvement work (O). Finally, participants felt that they had become more creative and critical thinkers since engaging with LSS education and training (O).

**Figure 7:**
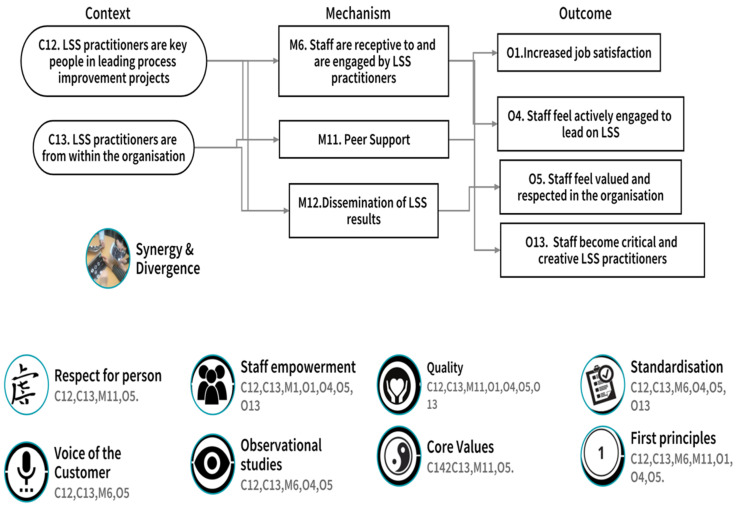
CMOc3 participants’ self-perception as LSS practitioners.

Figure 7 illustrates the contextual factors (Context), Mechanisms, and Outcomes that were confirmed, refuted, or refined through participant adjudication [35]. The previously identified synergy and divergence between LSS and person-centred care [18] are also mapped to the bottom of Figure 7. Finally, each of the noted synergy, divergence, or influence, relating to CMOc3 ‘Participants’ self-perception as LSS practitioners’ are numbered and therefore associated to the relevant Context, Mechanism, and Outcome. This allows us to visualise the alignment of CMOc3 to the synergy/divergence model (Figure 3) and reflects where study participants, through CMOc adjudication, aligned individual contexts, mechanisms, and outcomes to the identified synergy, divergence, or influencer.

As outlined in our methods, a review of the results of study participants’ LSS projects provided supporting evidence for the outcomes of their LSS work in their practice areas of improved patient and staff experiences, and patient outcomes. This was further supported by participants’ publications detailing the outcomes of their LSS practice [80,81,82,83,87,88,89,90,91,92].

We now, with reference to the relevant literature, discuss the alignment of the three CMOc from the realist evaluation (Figure 5, Figure 6 and Figure 7) to the synergy and divergence model (Figure 3) in order to clearly articulate the contribution of LSS to person cultures.

## 4. Discussion

Within this study the approach to the use of LSS within the study site was seen to be synergistic with the concepts of respect for persons and staff empowerment, themselves seen as enablers of person-centred cultures [30]. Joosten and colleagues [93] note that the development of staff through support and respect is important for their engagement with LSS, and participants advised they felt both supported and respected.

The approach of giving to employees through opportunities for development through the LSS education and training programme, rather than getting something from them, such as more productivity [94], is synergistic with the person-centred value of respect for persons, which is enabled by empowering cultures [30]. Joosten [93] notes the importance of linking process improvement to respect for the individual. Participants found that in their experience, LSS is synergistic with the concept of respect inherent in Lean [95] and the person-centred concept of respect for persons [96]. The focus of the LSS education and training programme on both patients’ and staff’s experiences of improvement was seen by participants as synergistic with respect for persons, as empowering staff and recognising the need for what we found in the literature are a principles and values-based approach to improvement [97].

Participants found that LSS enabled them to engage with people, using LSS tools to hear their voices [98] and, in keeping with LSS goals, to try to meet their expectations as customers [99,100]. This was facilitated by the use of Lean and Six Sigma quality tools designed to map the customer voice [5,6,101]. The LSS voice of the customer approach to understanding customers’ requirements is synergistic with person-centred care practices, which utilise observations, narratives, conversations, focus groups, and workshops [52]. The significance of open and clear communication for participants, clearly communicating the benefits of LSS work at the level of the department, unit, ward, or practice area, was found to be a cornerstone of their LSS practice. This is supported in the literature by the call for more open communication with staff [102] and is synergistic with the concepts of the voice of the customer. The use of LSS methodology actively sought to capture practitioners’ and their colleagues’ input, which has been demonstrated to give staff a voice in the nature and direction of improvement projects, and empowering them [103].

At the study site, it was found that staff were open to new ways of working when there was an integrative and distributive approach to LSS that was well communicated and supported by the availability of competent and accessible LSS practitioners. These factors contributed to a culture of empowerment and employee motivation, which Laloux [104] claims build consensus on the most effective use of Lean. These factors can also be seen as ‘humanising’ process improvement, which is a key context for any successful improvement process [98]. They also acknowledge the essential requirement of active staff engagement and empowerment in any quality improvement strategy. Staff empowerment and an organisational culture that encourages improvement are cornerstones of Lean deployment in healthcare [105] and we argue are synergistic with person-centred cultures that encourage and enable staff to engage in ongoing development and quality enhancement [106]. The presence of these factors also reflects the concept of ‘Kaizen’ and its origins in the three main features of the Japanese management philosophy: harmony and loyalty, consensus in decision-making, and employment for life [97].

Part of the local LSS intervention included practitioners of LSS at the study site visiting the areas where the process or work takes place, and standing back and observing the work or process, known in LSS as ‘Gemba’ [107]. Participants found this to be beneficial to their LSS practice. A Gemba walk is always approached from a place of mutual respect and of making thinking better and has been identified as being synergistic with the use of observational studies in person-centred care and person-centred cultures [18,102]. The processes involved in both approaches to these real-time observations of people at work [108] are virtually identical, with observational studies that Wilson and colleagues [109] outlined used within person-centred cultures to capture workplace culture.

From the synergy and divergence model (Figure 3), quality was identified as an influencer on both person-centred care and cultures, and LSS. Participants identified the approach to LSS work at the study site as having an integrative and distributive approach, which was leading to a more person-centred culture. The LSS education and training programme had contributed to this integrative and distributive approach to continuous improvement that sought to understand what staff, patients, and their families considered to be important in care delivery; that is, what was, in LSS terms, critical to quality [67]. This is consistent with participants’ views that the study site is receptive to person-centred approaches to care, and, through the LSS education and training programme, they are coming to recognise, as McCormack [29] found, that measurement and metrics are not the only, or even most important, components of a quality culture.

The synergy and divergence model identified that value can be seen in a wider context in person-centred care with a focus on patients, families, and staff and social values, whereas Lean focuses on the value created by improving processes [16]. During the realist evaluation, participants acknowledged this difference in the understanding of value and the divergence that exists. However, it was found that participants’ personal understanding as LSS practitioners echoed the wider context of valuing people and appreciating their core values as opposed to the narrower perception of value as that arising only from a particular process improvement, which participants indicated was the view of some staff and colleagues at the study site. Participants’ understanding of value can potentially more closely align their LSS practice to the first principles of person-centred care that seek to clarify people’s beliefs and values [16]. This broader understanding of value goes some way to addressing the divergence in the first principles and core values of LSS and person centredness, and to any potential lack of fidelity to Lean’s original principles, steps, intent, and purpose [13,64,110,111,112,113].

A potential for rigid insistence on standardisation when using LSS may be reflected in the variation in the use of the principles and steps of Lean [113], the use of a specific small set of tools or techniques [110], and/or the variation in Lean application [13]. Participants agreed that there was a dichotomy between the need for process standardisation and their wish to deliver more holistic individualised care. However, staff felt that the LSS education and training programme facilitated the ongoing reconciliation of this divergence in understanding the role of standardisation by enabling them to become proficient in the theory and practice of improvement methodologies, which is shown in the literature as leading to staff recognition of and appreciation for the requirement for process variations [114], when such variation may be in the patient’s best interests. Participants indicated that they had achieved outcomes that enabled them and their colleagues to further their professional development, spend more time with their patients, and work together as empowered and valued teams of practitioners in collaborative, inclusive, and participatory ways [9,84,104,115] to plan future improvements. Not only did staff perceive this but we have evidence from published findings of participants’ LSS work to suggest that those perceptions (reasonings) were on the back of a positive experience of engaging in LSS and have fed through into tangible patient or service outcomes [80,81,82,83,84,87,88,89,90,91]. Participants felt that the LSS education and training programme enabled practitioners using LSS and person-centred methodologies to provide person-centred, holistic, and individualised care, judging when patient care requires diversity while recognising that process standardisation can be useful and benefit patient outcomes [88].

The realist review illustrated how Lean does not fully consider the complexity of social interactions and dynamics in healthcare settings [93,116]. In the realist evaluation, participants confirmed the divergence between the concept of understanding value as a first principle of Lean and the imperative of person-centred care to attend to professional competence, to commit to ethical practice, and to clarify beliefs and values [16]. Participants identified gaps between colleagues’ understanding of the first principles of both LSS, and how they relate to person-centred care and cultures. This again demonstrates a lack of awareness of Lean as a management philosophy as opposed to a set of quality improvement tools [13,64,110,111,112,113,114]. Participants’ felt that their own presence as competent LSS practitioners from the organisation was as an important way to reconcile this divergence. Participants felt that, by developing staff versed in the theory and practice of improvement methodologies [114], the LSS education and training programme facilitated a move from a more technical to a more person-centred approach to change.

The initial programme theory of this study was that LSS can have a positive influence on person-centred care and person-centred cultures if delivered through the intervention of the LSS education and training programme. The alignment of the developed CMOc (Figure 4, Figure 5 and Figure 6) to the synergy and divergence model (Figure 3) indicates that in relation to the intervention of the LSS education and training programme, participants were able to identify:

- Contextual factors (C) that facilitated or hindered their LSS practice work.
- The outcomes (O) that emerged because of the action of underlying mechanisms (M), which they identified that were active when the contextual factors (C) were present.
- The synergies, influencers, and divergences between their LSS practice and person-centred care and cultures.

Prior to this study, little research was found on the complexities of introducing LSS into healthcare contexts in which the use of person-centred approaches is increasing. As a result, the contexts and mechanisms in and through which LSS and training programmes influence healthcare staff and person-centred practice were poorly understood. Based on the findings of the realist review and realist evaluation, we developed three CMOcs: LSS and aspects of organisational culture, the organisation’s receptivity to LSS, and participants’ self-perception as LSS practitioners, that resulted in an understanding of whether, how, and in what ways the intervention of an LSS education and training programme worked in diverse practice areas in the study site. Our previous work [18] identified four synergies and three divergences between LSS and person centredness as well as one influencer (Figure 3) that influenced both methodologies. These were aligned to the three CMOcs developed in the realist evaluation, further resulting in an increased understanding of whether, how, and in what ways the LSS education and training programme influenced person-centred practices and cultures. This alignment speaks to an organisation that is receptive to new and creative ways of working, and to an innovative model of LSS that can enhance efficiency and develop person-centred cultures [18]. This model of LSS also encourages staff self-development [32,66,93,117], developing employees through organisational support, respect, and access to education [118,119,120]. It enables LSS practitioners to address and reconcile divergences between LSS and person-centred practice in relation to core values and first principles and as such maximize patient and staff benefit from engaging in LSS projects. This is a model for improvement that recognises the benefit of combining LSS with the principles of person centredness to achieve efficiency and to preserve the autonomy and rights of staff [117,120], patients, and families. The current LSS education and training programme delivered to participants of the study site is a model for LSS practice that facilitates a culture of empowerment and is synergistic with person centredness [96] and makes a contribution to person-centred cultures. This model of LSS has as its basis an understanding that LSS is more than a set of quality improvement tools and techniques [13,64,110,111,112,113]. Rather it recognises that the intent of LSS-based improvement is to firstly value people, seeking to clarify their beliefs and values [16], and shows an understanding of Lean as a philosophy of life [120,121,122].

Our findings have also shown that LSS fails to contribute to person-centred cultures when there is divergence from people’s first principle at the level of LSS implementation. This is particularly evident where it has been wrenched from its original purpose and underpinning philosophy and focuses only on process standardisation and efficiency gains, with even then wide variance in the application of the implementation of Lean [13,110]. This situation arises when LSS practitioners are unaware of, pay little attention to, or fail to understand Lean’s philosophical roots [13,18,64,110,111,112,113,122]. This failure to recognise, or to have fidelity to [113] Lean’s philosophical roots and its original intention to clarify people’s beliefs and values [16] as a prerequisite step to improvement is an important finding with implications for theory, research, education, policy, and practice. Knowledge of Lean’s philosophical roots, with its focus on valuing people and their values from the start, was, however, shown to have synergies with the philosophical intentions of person centredness [18]. Our previous work [18] identified that coherence in philosophy, intention, methods, and outcomes exists between the LSS and person-centred approaches in healthcare. This study’s realist review and subsequent realist evaluation have shown that this coherence is present in the study site. This knowledge of the synergies that exist between both LSS and person-centred methodologies and how their divergences may be reconciled could impact the design and direction of further theory, education, and research in this area and inform future healthcare improvement policy. Importantly, it could also enable LSS practitioners internationally to work in ways that support the development of quality, person-centred care that takes account of the outcomes for, and experiences of, patients, their families, and staff.

This study was not without limitations. There was a lack of published research specific to the use of both person-centred and LSS methodologies to draw on for the realist review that underpinned this study; however, the initial programme theory was informed not only by the literature but also through engagement with the expert panel. Only one education programme was evaluated, a particular university LSS education and training programme, which was developed over the last six years and is likely to be quite different in its design and development from other LSS education programmes. However, the study findings offer opportunities for reflection, learning, and development for other providers of LSS education that needs to be further evaluated to understand its overall relevance and impact in a range of contexts. There was constant awareness throughout the realist evaluation of our primary researcher being an insider researcher and of the potential influence on the research and on the willingness of others to participate. We acknowledged and discussed this positionality and used reflexivity within this study to ensure responsible and ethical practice throughout the research process. The use of researcher reflexivity enabled engagement in critical self-reflection about any personal biases, preferences, and preconceptions [123]. Additionally, the use of reflexivity throughout the study is consistent with best practice for insider researchers that recommends critical reflection is included in the study design [124]. Although being an insider is here recognised as a limitation, knowing the culture and context of the study site did support the provision of a psychologically safe environment for participants.

## 5. Conclusions

Wilson and McCormack [125] suggest that the evaluation of an intervention (in this case, an LSS education and training programme) enables the uncovering and analysis of the causal mechanisms operating at the level of the real. Pawson states that this enables the realist researcher to “look beneath the surface in order to inspect how they [causal mechanisms] work” [33] (p. 24). In this study, participants were clear that the intervention of an LSS education and training programme had contributed to what is termed a ‘culture of quality’ [126] in their organisation. This is congruent with the argument that LSS deployment is not just about the quality improvement itself [127] but about creating a supportive institutional culture [9,72,128]. It is also synergistic with the cultural aspect of person centredness that promotes and incorporates care [106]. This study identified coherence in the underlying philosophy, intention, method, and outcomes of LSS and person-centred approaches to process improvement and highlights the positive impact an integrated (LSS and person centred) approach to facilitation of LSS on both patient outcomes and health care culture. This contributes to researchers’, policy makers’, and practitioners’ awareness and understanding of the origins, purpose, and methods of LSS and should inform its introduction and implementation in practice. Whether or not such an understanding exists will determine its impact on the further development of person-centred care and cultures across health systems internationally.

## Data Availability

The data presented in this study are available in the paper.
